# Copper Amalgam

**Published:** 1889-11

**Authors:** Julian W. Russell

**Affiliations:** Brooklyn, New York


### COPPER AMALGAM.[1]

BY JULIAN W. RUSSELL, M.D.S., BROOKLYN, NEW YORK.

THE rapid rise of copper amalgam in this country during the past eighteen months has been simply marvellous. In order to show how great this is, I have endeavored to gather all the facts possible in regard to the quantity used. Two years ago there were not more than two hundred ounces used per year in this country. Last year the consumption was about four thousand ounces, and this year the demand will approach ten thousand, or nearly ten per cent. of the total amount of alloy and amalgam consumed in the United States. Though used abroad for nearly forty years, it seems singular that so little should have been employed in this country until so recently. The craze for it—for we may call it so—has been equalled only by that of cocaine of three years back, though with this difference, the demand for cocaine rapidly diminished, and that drug is now used in comparatively few offices. The yearly demand for copper amalgam, however, will not increase much more than the amount that I have estimated, neither will it diminish, but will, I think, hardly change for years to come; for though some have abandoned, and others will abandon, its use, yet there are still others who will take it up, and so the demand will remain about the same.

Unfortunately the craze for copper amalgam, like some of the other fads that have afflicted the dental profession during the past few years, has caused this material to be employed to excess in a great many cases; and, though some of its most ardent admirers

---

[1] Read before the New Jersey State Dental Society, at its nineteenth annual session at Asbury Park, July 18, 1889.

maintain that it is the only material to use, yet during the next year we shall hear of so many failures that even these will have to change their minds, and admit that it must be used with discretion, and the word "discretion" is the one that I wish particularly to emphasize. Copper amalgam, employed with discretion, is undoubtedly one of the best materials that the dentist can make use of to preserve the teeth. But how many employ it in that way? Very few, as yet, I regret to say. Their own failures, however, will soon teach them, and they will in the course of time understand the meaning of the word discretion.

It is a curious fact, and one that illustrates the great difference between this country and Europe, that though they have been manufacturing amalgams for so many years, yet the material they make abroad to-day is precisely the same that they made forty years ago. And whilst the American amalgams are not yet two years old, yet the improvement in their manufacture has been so great that we can now export them to the countries of their birth, though sold at a far higher price. Let any one try the various amalgams and note the vast difference between the foreign and the domestic. The foreign article is, as a rule, very dirty, coarse-grained, and contains about seventy-five per cent. of mercury; and whilst some of the American are precisely like these, yet there are others which are as clean and pure as it is possible to make them. This is especially true of those that are made by electricity. Though there are some amalgams which contain a large surplus of mercury, there are also others which have only about twenty-five per cent. of this substance.

The old method of manufacture was by precipitating the copper from its solution of sulphate, either by means of iron or zinc plates or bars. There was no way of controlling the size of the grains of copper, and the result was that some would be very fine, whilst another lot would be as coarse as white sand.

The newest and best method is precipitating by electricity generated by a dynamo. By regulating the current the resulting amalgam may be made so fine that in rubbing it between the fingers it will feel like satin. The plant is very expensive, and requires an expert electrician to run it. But in time, I think, all copper amalgam will be made in this manner, as the resulting product is chemically pure.

Some time ago an article on copper amalgam, written by Dr. St. George Elliott, of London, was extensively copied by the journals in this country. He gave the results of a number of experiments made by him with alloys and copper amalgams, the experiments not resulting favorably to copper amalgam. The great mistake he made, I think, was in beating the amalgam until some of the copper was oxidized ; this would result in making it very brittle, and so it could not compare at all favorably with the alloys which he tested at the same time. Another was that the article was coarse-grained, and hence these experiments are of little service to us who use an entirely different article.

He also said that around a great many fillings the tooth-substance would soften. I have noticed this only when the amalgam was made by what is called the zinc process. In making it in this manner a certain percentage of zinc is unavoidably incorporated into the amalgam, and when placed in the tooth and moistened with saliva it seems to generate a small quantity of electricity, the filling acting as one pole of the battery, and the tooth the other. The result is a gradual dissolution of the tooth-substance around the margin of the filling.

Iron as an impurity does not appear to give this result. Zinc has also the property of making the amalgam quick-setting. A certain test for it is to mix up a quantity of the amalgam with a considerable surplus of mercury, making the mass so soft that it will not retain its shape when rolled into a ball. If this should get hard in the course of two or three hours, it denotes the presence of zinc. If there is no zinc, the mass will remain soft for a whole day.

I have heard many believers in this material assert that many of their fillings do not tarnish, but keep a bright silver color, like an alloy. These are the very cases that will result in failures in the course of time, for a copper amalgam filling retaining its brightness is a sure sign of a highly acid condition of the secretions, and these fillings are slowly dissolving. If examined after a few months, they will be found scooped out in all directions, exposing the margins of the cavity and forming a recess for fresh decay. The only remedy for this is to remove a portion and refill with alloy or gold. When, on the contrary, the filling turns black or dark brown, then it can be left alone with perfect safety, and with the knowledge on the part of the operator that it will preserve the tooth for many years.

Dr. Rollins, of Boston, recently related to me a case in which he inserted a filling that weighed forty grains; two years afterwards he removed it and found that it weighed only twenty grains. The rest had been dissolved by the acid secretions. When an operator desires to fill a large number of cavities in one mouth, the best plan to pursue is first to insert one filling, and let it remain two or three days; if at the end of that time the filling has turned a dark color, the remaining fillings can then be inserted; but if, on the contrary, it still remains bright, he had better use some other material. The best places to use this material are in the deciduous, the wisdom teeth, and in teeth of a soft chalky character, where nearly all other filling materials fail to preserve them. In filling deciduous teeth it is not necessary to remove more than the decay around the margin; thus the danger of exposed pulps is avoided, and, as amalgam is less of a conductor than alloy, it can be placed in very sensitive cavities without causing irritation. One of its peculiarities is the perfect adaptation to the walls of the cavity, neither shrinking nor bulging; and cavities under the margin of the gum can be smoothed so thoroughly with a burnisher that a subsequent finishing is unnecessary. It is particularly adapted to cavities in the buccal surfaces of the molars. Especially is this the case with wisdom-teeth, which the brush seldom reaches, and which, when filled with alloy, decay rapidly around the margins. Where it is impossible to keep the cavity dry, amalgam can also be used, only then it will darken the tooth, from the oxide formed by the moisture. In dry cavities, however, there is no discoloration, and this allows it to be placed behind thin walls of enamel. It is also useful in repairing old fillings of gold or alloy, where they are undermined by decay, but are too good to be removed. Crowns can be moulded from it, and fastened to roots by means of pins and a thin mixture of amalgam. A number of operators have said that it is impossible to join an alloy and an amalgam filling when they are soft. Some alloys will join very readily, as I have proved by filling one-half a glass tube with alloy and the remainder with copper amalgam. When hard, the tube was broken, and it was impossible to break them apart at the joint.

Copper amalgam is also useful in combination with gold at the cervical wall, but it is preferable first to fill that part with the amalgam, allowing it to harden for a day, then drill your retaining points and fill with gold. Where the dark color is an objection to copper amalgam, a few pieces of gold can be burnished over the surface when it is soft. The gold will take up the surplus mercury, and when finally finished the filling will be of a gold bronze color.

In preparing this filling for the cavity, take as dry an amalgam as possible, heat it slightly until the mercury just appears on the surface, place in a mortar, and grind very rapidly. The friction will soon render the mass very soft. Place in the palm of the hand and pat it gently until the whole mass is amalgamated. Sometimes it may be necessary to warm it again. If wanted to set rapidly, squeeze through a napkin with a pair of pliers, for the more mercury extracted the quicker it will harden. More care is required in filling cavities with this material than with alloy, but when used with the precautions that I have indicated the results are very satisfactory.

In conclusion, as to the future of copper amalgam, I believe that it is an excellent filling material, and that in time it will be found in the office of every careful, conservative dentist.

